# The Impact of Metformin on BNP Levels: A Potential Cardioprotective Role in Type 2 Diabetes

**DOI:** 10.3390/jcm14082733

**Published:** 2025-04-16

**Authors:** Emre Hoca, Nilsu Kalaycı, Süleyman Ahbab, İsmail Engin, Hayriye Esra Ataoğlu

**Affiliations:** 1 Department of Internal Medicine, Haseki Training and Research Hospital, University of Health Sciences, 34260 Istanbul, Turkey; dr.nilsukalayci@gmail.com (N.K.); drsahbab@gmail.com (S.A.); eataoglu@gmail.com (H.E.A.); 2 Department of Endocrinology, Ümraniye Training and Research Hospital, University of Health Sciences, 34760 Istanbul, Turkey; ismailengin1987@gmail.com

**Keywords:** brain natriuretic peptide, cardiovascular complications, heart failure, metformin, type 2 diabetes

## Abstract

**Background/Objectives**: Cardiovascular complications are the most common cause of mortality and morbidity in diabetic patients. Therefore, the aim of antidiabetic therapy should not only be to provide glucose regulation but also to protect patients from complications and related mortality. Brain natriuretic peptide (BNP) is a peptide secreted as a result of myocardial stress. BNP levels increase under conditions of increased myocardial stress, such as heart failure. It is an important marker not only at the time of diagnosis but also during follow-up. In our study, we aimed to evaluate BNP levels and thus, the factors affecting the risk of developing heart failure during the course of diabetes. **Methods**: This study was conducted at the diabetes outpatient clinic of the University of Health Sciences, Haseki Training and Research Hospital. A total of 252 patients met the inclusion criteria and were enrolled in the study. All study participants were patients with a confirmed diagnosis of type 2 diabetes. Laboratory parameters, including BNP values, comorbidities, and anamnesis data, were recorded. **Results**: The mean BNP levels were significantly lower in patients using metformin and pioglitazone. Other antidiabetic medications were not associated with BNP levels. BNP levels were positively correlated with age and diabetes duration and negatively correlated with hemoglobin levels. According to regression analysis, age, metformin use, and hemoglobin levels were found to independently affect BNP levels. **Conclusions**: Our findings suggest that metformin could potentially play a significant role in preventing the development of heart failure in diabetic patients currently not experiencing this complication owing to its favorable effects on myocardial stress. This suggests metformin’s potential in preventing heart failure in type 2 diabetic patients.

## 1. Introduction

Cardiovascular diseases (CVD) continue to be the most common cause of morbidity and mortality during the course of type 2 diabetes (T2D) [1]. Insulin resistance as a main pathophysiologic cause of T2D is associated with an atherogenic lipid profile, endothelial dysfunction, and increased abdominal obesity, which significantly increase the risk of cardiovascular events [2,3]. Among individuals with type 2 diabetes, the risk of developing CVD is markedly higher due to shared pathological mechanisms, including chronic inflammation, oxidative stress, and endothelial dysfunction [4]. Heart failure, with a prevalence of up to 30%, is among the most common cardiovascular complications in diabetic patients, together with myocardial infarction [5,6]. Also, even in the absence of overt heart failure, subtle changes in cardiac structure or function may go unnoticed for years. Identifying biomarkers that predict cardiovascular risk and understanding the impact of therapeutic interventions on these biomarkers are paramount. Brain natriuretic peptide (BNP), which is produced from pre-proBNP and released from myocytes under myocardial stress and stretch, is a well-established biomarker of heart failure [7]. BNP and N-terminal (NT)-proBNP are both natriuretic peptides, but the superiority of one over the other in the prediction of heart failure has not yet been demonstrated. Both peptides are actively used in the diagnosis and follow-up of heart failure [8]. A BNP level below 100 is considered normal, while a level above 400 indicates the probability of heart failure. Values between 100 and 400 are considered a “gray zone”, and heart failure is evaluated according to symptoms, examination findings, and clinical data [9,10]. Elevated levels of BNP have also been associated with subclinical cardiovascular dysfunction and increased cardiovascular risk in non-heart failure populations, including patients with T2D [11]. Therefore, studies show that BNP levels can be used to monitor heart failure and evaluate its prognostic importance in diabetic patients [12,13]. However, the factors influencing BNP levels in individuals with T2D who do not exhibit overt heart failure remain poorly understood.

Metformin is the most commonly used, first-line antidiabetic agent employed to improve insulin sensitivity in treating type 2 diabetes and conditions characterized by insulin resistance, such as pre-diabetes, obesity, and polycystic ovary syndrome [14]. Metformin also displays advantages over other antidiabetic agents, such as a favorable safety profile and low cost. Studies show that metformin contributes favorably to mitochondrial energy metabolism in cardiac cells by activating AMP-dependent kinase (AMPK). Some other studies have shown that metformin can reduce oxidative stress in cardiac cells, contributing to an increase in endothelial function and positively affecting inflammation [15,16]. There are very few studies evaluating the beneficial effects of metformin on the development of heart failure in diabetic patients or comparing its cardiac effect profile head-to-head with those of agents such as sodium-glucose cotransporter-2 (SGLT2) inhibitors and glucagon-like peptide-1 (GLP-1) receptor agonists, whose positive cardiovascular effects have been well established. In addition, very few studies evaluate the effects of metformin on cardiac stress via BNP levels.

In this retrospective cross-sectional study, we aimed to investigate the effects of antidiabetic drugs on cardiac stress and their possible cardioprotective effects in diabetic patients without heart failure by evaluating the measured BNP levels. In addition to comparing BNP levels among different antidiabetic treatments, we also aimed to explore whether metformin use was associated with more favorable cardiac biomarker profiles and possible cardioprotective effects in patients without overt heart failure.

## 2. Patients and Methods

### 2.1. Study Participants

The study protocol was approved by the ethics committee of Haseki Training and Research Hospital, Istanbul, Turkey (reference no: 58-2024, date: 1 August 2024). This study was conducted using the principles of good clinical practice and according to the Declaration of Helsinki. The data of patients who attended the diabetes outpatient clinic between 1 January 2024 and 1 October 2024 were retrospectively reviewed. A total of 6299 patients were screened, and those meeting the inclusion criteria (*n*: 252) were enrolled in the study. Patient and laboratory data were collected and analyzed using the hospital information operating system. Being older than 18 years of age, having a BNP level below 400 pg/mL, being diagnosed with type 2 diabetes, and having accessibility to the anamnesis of the treatments used by the patients were determined as inclusion criteria. Exclusion criteria included a BNP level above 400 pg/mL suggestive of heart failure, echocardiographically proven heart failure, or the presence of symptoms of heart failure (New York Heart Association Heart Failure Functional Classification class II, III, IV patients). Patients with chronic kidney disease (CKD) stage 4–5, acute kidney injury, or hepatic failure were also excluded. SGLT2 inhibitors, DPP-4 inhibitors, sulfonylureas, and insulin were commonly used as adjunctive therapies in combination with metformin. A small number of patients were using GLP-1 receptor agonists; however, due to their low representation in the sample, they were not included in the final analysis. Most patients using metformin received standard oral doses between 1000–2000 mg/day. Due to the retrospective nature of the study, specific information on dose titration and treatment duration was incomplete.

### 2.2. Data Collection

Demographic data (such as age and gender) and clinical characteristics (comorbidities and medications used) were recorded. The laboratory parameters (BNP, aspartate aminotransferase [AST], alanine aminotransferase [ALT], triglycerides, high-density lipoprotein [HDL] cholesterol, low-density lipoprotein [LDL] cholesterol, total cholesterol, complete blood count, HbA1c, glucose, urea, creatinine) obtained following an overnight fasting period of at least 8 h were recorded. Diabetes duration was calculated and recorded by analyzing the anamnesis, medication usage reports, and patients’ past prescriptions. In order to complete the missing data, the hospital information operating system was used. BNP levels were determined using a chemiluminescence immunoassay (CLIA) method with a BNP kit via a Siemens Advia Centaur XP (Mannheim, Germany) device and recorded in nanograms per liter.

### 2.3. Statistical Analysis

Statistical analyses were performed using SPSS 25.0 for Windows software (IBM Corp., Armonk, NY, USA). Descriptive statistics were presented as mean ± standard deviation and minimum–maximum and median values. The normality of the continuous variables was assessed using the Kolmogorov–Smirnov test. Comparisons between groups were conducted using the independent samples t-test or the Mann–Whitney U test, depending on relevant data distribution. Categorical variables were compared using the Chi-squared test. Correlations between BNP levels and clinical or laboratory parameters were evaluated using Pearson or Spearman correlation coefficients. Patients were retrospectively categorized as “user” and “non-user”, according to medication use, for subgroup analyses. Patients with comorbidities were typically being treated with related pharmacotherapies (e.g., statins, antihypertensives). Although these were not all included in the regression model, comparisons were made, where relevant. In multivariate analysis, we included antidiabetic agents such as metformin and pioglitazone to explore their independent associations with BNP levels. Different multivariate linear regression models were constructed to identify independent factors associated with BNP levels. While constructing different regression models, in addition to the data that we found to be associated with BNP in our study, some other parameters found to be associated with BNP in previous studies were also used. Model 1 included demographic and treatment-related variables (age, gender, metformin or pioglitazone use). Model 2 additionally included laboratory markers such as HbA1c, hemoglobin, and the lipid profile to assess the independent contribution of each factor to BNP levels. A *p*-value of <0.05 was considered statistically significant.

## 3. Results

A total of 252 patients meeting the inclusion criteria were included in the analysis. The mean age of the study population was 56.82 ± 9.2 years, with a diabetes duration of 9.96 ± 5.04 years. Of the participants, 132 were female and 120 were male. The mean BNP level was 37.43 ± 39.59 pg/mL. Since the BNP values were not normally distributed and the standard deviation was high, we calculated the interquartile range (IQR 25–75%: 13.65–46.55), along with the median level (25.7). Non-parametric methods, such as the Mann–Whitney U test, were used to analyze the BNP levels. The mean HbA1c level was 7.90 ± 1.77%, and the mean fasting glucose level was 152.24 ± 59.59 mg/dL. The lipid profile results showed a mean total cholesterol level of 176.73 ± 42.13 mg/dL, HDL cholesterol of 43.64 ± 12.05 mg/dL, LDL cholesterol of 101.58 ± 35.52 mg/dL, and triglycerides of 160.45 ± 94.06 mg/dL. The mean hemoglobin (Hb), urea, and creatinine levels were 13.55 ± 1.58 g/dL, 35.03 ± 11.26 mg/dL, and 0.90 ± 0.22 mg/dL, respectively (Table 1). Comorbidities with diabetes included hyperlipidemia in 51%, hypertension in 41%, coronary artery disease in 10%, and hypothyroidism in 5% of patients. (Table 1).

**Figure 1:**
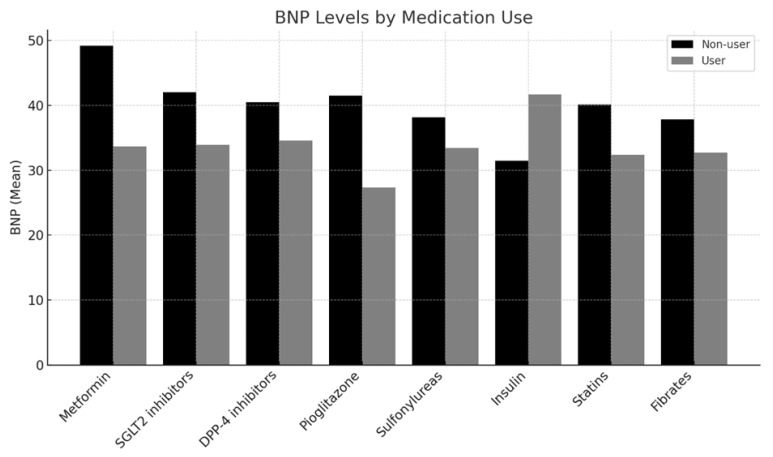
BNP levels by medication.

The mean BNP levels were significantly lower in patients using metformin (33.68 ± 36.78 pg/mL) than in non-users (49.18 ± 45.69 pg/mL; *p =* 0.034). Similarly, pioglitazone users revealed significantly lower BNP levels (27.37 ± 22.85 pg/mL) than those of non-users (41.46 ± 43.98 pg/mL; *p =* 0.021). However, no significant differences in BNP levels were observed for users versus non-users of SGLT2 inhibitors, DPP-4 inhibitors, sulfonylureas, insulin, statins, or fibrates, as shown in Table 2 and Figure 1. Among the 87 patients receiving statin therapy, 77 (88.5%) were also using metformin. However, statin use did not result in a statistically significant difference in BNP levels.

A significant positive correlation was found between BNP levels and age (r = 0.410, *p* = <0.001) as well as diabetes duration (r = 0.149, *p* = 0.018). BNP levels showed a negative correlation with hemoglobin (r = −0.284, *p* = <0.001) and alanine aminotransferase (ALT) levels (r = −0.151, *p* = 0.016). No significant correlations were observed between BNP levels and HbA1c, fasting glucose, lipid profile, urea, creatinine, or AST levels (Table 3).

When subgroup analyses were performed, the BNP level was found to be 44.77 ± 47.52 in females and 29.35 ± 26.36 in males (*p* = 0.001). There was no difference between the mean ages of male and female patients (57.23 ± 8.82 and 56.37 ± 9.60, respectively, *p* = 0.459).

The two-step regression model approach was used to test the robustness of associations by progressively adjusting for more variables. Although various drug groups including statins, insulin, SGLT2 inhibitors, and DPP-4 inhibitors were evaluated in univariate analyses, only metformin and pioglitazone showed statistically significant associations with BNP levels and were included in the regression models. Multivariate linear regression analysis (model 1) identified age (B: 1.305, *p* = <0.001), male gender (B: −12.915, *p =* 0.006), and metformin use (B = −11.312, *p* = 0.040) as independent predictors of BNP levels. In an alternative regression model (model 2), age (B: 1.293, *p =* <0.001), metformin use (B: −11.857, *p =* 0.047), as well as hemoglobin (B: −3.980, *p =* 0.020), were found to be significant predictors. Pioglitazone use, HbA1c, LDL cholesterol, HDL cholesterol, and creatinine were not significant predictors of BNP levels, according to these regression models, as shown in Table 4.

## 4. Discussion

The aim of the treatment of diabetic patients should not only be to reduce blood glucose and HbA1c levels but also to prevent possible complications and serious morbidity and even mortality that may develop as a result of these complications [17,18]. Since cardiovascular events are the most common cause of death among these complications, favorable cardiovascular effects are at the forefront when developing diabetes treatments or evaluating the effects of existing antidiabetic agents. Considering that all diabetic patients are at risk for cardiovascular diseases, we aimed to evaluate the factors affecting BNP levels, which are indicators of cardiac stress, and thus the risk of developing heart failure in diabetic patients without a diagnosis or symptoms of heart failure. Thus, we demonstrated that BNP levels may be an important indicator in the selection of an antidiabetic treatment. In our study, among all antidiabetic drugs, only metformin displayed a decreasing effect on BNP levels, making metformin the first agent of choice in the treatment of diabetic patients at risk of cardiovascular disease.

We included patients with BNP values up to 400 pg/mL, which made it possible to evaluate cases with borderline or mildly elevated levels. This reflects a patient group often encountered in daily practice, especially in those with type 2 diabetes who have not been diagnosed heart failure. Furthermore, our study includes a large sample and compares the effects of several antidiabetic agents on BNP levels using multivariate regression models.

Hyperlipidemia was the most common comorbidity in our patient group. Although we did not collect detailed anthropometric data, such as body mass index or waist circumference, clinical experience and the existing literature suggest that a considerable portion of these patients may also have been obese. The coexistence of these conditions could have contributed to the overall cardiometabolic burden, potentially influencing BNP levels.

BNP levels increase with age due to impaired ventricular compliance, endothelial dysfunction, and increased preload and afterload due to the increased activity of the renin–angiotensin–aldosterone system. Therefore, studies suggest that age-specific cut-off values of BNP levels should be determined [19,20]. Similarly, there are studies showing that an increase in the duration of diabetes is also associated with an increase in BNP levels [21]. In our study, we found a positive correlation between both age and diabetes duration and BNP levels. Later, when we performed regression analysis, although age was found to be one of the factors independently affecting BNP levels, in the second model, it was observed that diabetes duration was not one of the factors independently affecting these levels. This finding suggests that the effect of diabetes duration on BNP levels may be due to other factors such as age and medications used.

Hypothyroidism can affect BNP levels via its effects on cardiac function. In our study, we did nott find a significant difference in BNP values between patients with and without hypothyroidism. Still, the small number of hypothyroid patients limits the strength of this comparison.

In the majority of studies, BNP levels were found to be higher in women than in men among patients without a diagnosis of heart failure. Although the underlying pathophysiology has not been fully elucidated, the main factors are thought to be that testosterone upregulates neprilysin activity and leads to a decrease in cardiac natriuretic peptide levels. At the same time, the estrogen hormone increases gene expression and the release of natriuretic peptides [22,23,24]. In our study, BNP levels were found to be significantly higher in female patients, supporting these findings. In addition to the possible pathophysiologies suggested, the fact that hemoglobin levels were lower in the female patient group and hemoglobin levels were negatively correlated with BNP, the lower hemoglobin levels in women mediated the possibility of BNP elevation. The second regression analysis model created with demographic data and laboratory parameters found that hemoglobin levels were an independent parameter affecting BNP levels, but gender had no effect on BNP. This suggests that the difference in BNP levels between genders may have arisen due to differences in hemoglobin levels. Low hemoglobin levels are thought to increase BNP levels by increasing the stress on the myocardium, leading to an increase in left ventricular wall thickness in later periods. In studies conducted by Wold Knudsen et al. and Karakoyun et al., an inverse relationship was observed between hemoglobin levels and BNP levels in different patient groups, with and without a diagnosis of heart failure [25,26]. Although we observed a negative correlation between hemoglobin and BNP levels, the underlying mechanism remains uncertain, particularly in patients without heart failure. This association may reflect subclinical myocardial strain or reduced oxygen-carrying capacity, but causality cannot be inferred.

While cholesterol levels are associated with processes resulting in atherosclerosis, BNP levels reflect the level of cardiac stress through different pathophysiological mechanisms, independent of atherosclerosis. As a result, there may be no correlation between cholesterol levels and BNP levels. Similar to the results of our study, no significant correlation was found between BNP levels and LDL cholesterol and HDL cholesterol levels in the study conducted by He et al. with elderly patients [27].

Srisawasdi et al. showed that BNP levels were less affected by creatinine levels than were NT-proBNP levels, and even in male patients, patients with stage 3 CKD had similar levels to those of men with stage 1 CKD when other factors were eliminated. This is thought to be due to the higher renal excretion of NT-proBNP compared to that of BNP. It has been shown that BNP levels usually start to be affected by creatinine levels in patients with advanced CKD (stage 4 or 5) [28]. Similarly, Vickery et al. demonstrated that the levels of natriuretic peptides, mostly NT-proBNP, started to be affected in advanced CKD patients [29]. As in the aforementioned studies, we found no correlation between creatinine levels and BNP levels in our patient groups. Our study excluded patients with advanced chronic kidney disease and used BNP levels as the primary natriuretic peptide marker.

A modest negative correlation between ALT and BNP levels was observed. Although the mechanism is unclear, it may reflect subclinical hepatic–metabolic interaction or liver-mediated regulation of natriuretic peptides.

Studies reveal that BNP levels in diabetic patients have a close relationship with glycemic control and that BNP levels decrease as HbA1c and glucose levels normalize; other studies show that BNP levels do not correlate with glycemic control and may even have a negative correlation, in some cases [30,31]. This may be because BNP protects against insulin resistance by increasing fat metabolism, increasing adiponectin levels, and decreasing inflammation [32,33]. Our study did not find any correlation between glucose or HbA1c levels and BNP levels. We thought that this was normal because the majority of the type 2 diabetes patients admitted to our diabetes outpatient clinic also exhibited concomitant metabolic syndrome. While metabolic syndrome may influence BNP dynamics in T2D, we lacked the specific diagnostic criteria (e.g., waist circumference, blood pressure) to confirm its presence in our cohort and therefore did not analyze it directly.

Most studies evaluating the cardioprotective effects, particularly changes in the natriuretic peptides of SGLT2 inhibitors, have been conducted in patients with heart failure. SGLT2 inhibitors have been found to cause a significant decrease in BNP in these patients. The study by Chen et al. found similar results [34]. On the other hand, there are very few studies evaluating the relationship between SGLT2 inhibitors and natriuretic peptides in patients without a diagnosis of heart failure or symptoms of heart failure. In the SOCOGAMI study and the EMBODY trial, the effect of SGLT2 inhibitors on cardiometabolic parameters in patients without a diagnosis of heart failure was evaluated, and it was found that SGLT2 inhibitors had no significant effect on BNP levels in these patients [35,36]. SGLT2 inhibitors act by decreasing congestion and preload in patients with heart failure, leading to a decrease in BNP levels as a result of decreased ventricular tension. Although SGLT2 inhibitors have demonstrated cardioprotective effects in both heart failure and non-heart failure patients—as shown in trials such as EMPA-REG OUTCOME and DAPA-HF—these benefits may not always be accompanied by significant changes in BNP levels, particularly in patients without overt cardiac dysfunction [37,38]. BNP is a practical marker for detecting subclinical myocardial stretch; however, it may not capture all aspects of cardiovascular benefits. In our study, the association between metformin use and lower BNP levels may suggest that metformin exerts a favorable effect on myocardial stress or metabolic status—potentially through mechanisms more directly reflected in BNP concentrations. Similarly, in our study, no significant difference was observed in BNP levels in patients using SGLT2 inhibitors compared to the levels for non-users.

It is thought that pioglitazone may increase BNP levels due to its water-retaining effect, thus possibly increasing myocardial volume [39]. In our study, BNP levels were found to be lower in the patient group using pioglitazone, which may contradict this finding. However, the regression analysis later found that pioglitazone use was not an independent factor affecting BNP levels. When we performed subgroup analysis, we found that the mean age of pioglitazone users was lower than that of non-users, and most of these patients were pioglitazone users.

Until recently, it was recommended that metformin should not be used in patients with heart failure due to the possible risk of lactic acidosis. In light of current information, it is thought that metformin can also be used in patients with heart failure, as well as in diabetic patients at risk of developing heart failure, due to its anti-inflammatory, myocardial oxidative stress-reducing, and endothelial function-correcting effects [16]. Moreover, some studies have shown that there is no significant increase in the risk of lactic acidosis with metformin use in patients with heart failure [40]. In addition to these favorable effects of metformin use, there are very few large-scale studies evaluating the relationship between BNP levels and metformin use in patients without a diagnosis of heart failure, and the results are contradictory. In the study by Top et al., no change was observed in BNP levels in the patient group using metformin, but we thought that the fact that all of the patients included in the study were using insulin might have caused no change in BNP values due to the effects of insulin [41]. In the study conducted by Fawzi et al. on non-diabetic patients diagnosed with heart failure, the addition of metformin to standard heart failure treatment resulted in a significant reduction in BNP levels during follow-up [42]. Similarly, Sokolova et al. observed that metformin use led to a decrease in BNP levels in patients with diabetes who were not experiencing heart failure [43]. Although both studies were conducted with a relatively small number of patients, they are valuable in demonstrating the potential cardioprotective effects of metformin. Consistently, our study also found that metformin use was associated with a significant reduction in BNP levels, independent of other factors. Metformin was frequently used alongside other antidiabetic medications, and our multivariate analysis showed that only metformin use independently predicted lower BNP levels, suggesting that the effect is not solely due to combination therapy. Although SGLT2 inhibitors and GLP-1 agonists have demonstrated significant cardiovascular benefits, metformin’s direct effect on BNP levels remains less explored. Metformin may influence BNP levels through mechanisms other than glucose control. These may involve AMPK activation, reduced oxidative stress, better endothelial function, and suppression of chronic low-grade inflammation. Together, such effects might ease myocardial wall tension and help lower BNP release. Our findings highlight a unique role for metformin in reducing myocardial stress, which could have implications for future diabetes treatment strategies. These findings suggest that, in addition to its beneficial effects on blood glucose regulation and insulin resistance, metformin may play an unexpectedly crucial cardioprotective role in diabetic patients.

The limitations of our study include the fact that it was retrospective and cross-sectional; we evaluated the status of the patients at the time of data collection, and we could not observe changes in the parameters during follow-up. Dose and duration data for metformin and other medications (e.g., antihypertensives) could not be uniformly collected, which limits the assessment of dose–response and medication–response relationships. We were unable to evaluate important confounding factors such as neprilysin activity, physical stress, systemic inflammation, and infection status, all of which may influence BNP levels.

## 5. Conclusions

Heart failure, one of the most common cardiovascular complications that may develop during the course of diabetes, is a pathology whose morbidity and mortality can be reduced if its development can be prevented. The positive effects of metformin use on BNP levels, and thus on the risk of developing heart failure, even in diabetic patients without heart failure, are quite valuable. If the benefits of metformin can be demonstrated in studies conducted with a larger patient group that include patients follow-up data, this under-emphasized effect of metformin could allow it to once again become the first choice for the treatment of diabetics, especially those at risk of developing heart failure.

## Figures and Tables

**Table 1:** Descriptive variables and laboratory parameters of patients with T2D.

Variable	Mean ± Std	Min.–Max. (Median)
Age (years)	56.82 ± 9.2	23–75 (58)
Diabetes duration (years)	9.96 ± 5.04	1–17 (12)
Gender (F/M)	132/120	
BNP (ng/L)	37.43 ± 39.59	2–314 (25.7)
HbA1c (%)	7.90 ± 1.77	4.8–15.5 (7.45)
Glucose (mg/dL)	152.24 ± 59.59	47–430 (137)
Total cholesterol (mg/dL)	176.73 ± 42.13	77–335 (174)
HDL cholesterol (mg/dL)	43.64 ± 12.05	16–95 (42)
LDL cholesterol (mg/dL)	101.58 ± 35.52	20–251 (94.8)
Triglycerides (mg/dL)	160.45 ± 94.06	37–602 (133.5)
Hemoglobin (g/dL)	13.55 ± 1.58	9.2–17.1 (13.5)
Urea (mg/dL)	35.03 ± 11.26	12–96 (33)
Creatinine (mg/dL)	0.90 ± 0.22	0.48–1.84 (0.87)
AST (U/L)	16.71 ± 14.59	4–188 (14)
ALT (U/L)	27.17 ± 19.19	8–160 (22)
**Comorbidities**	**N (%)**	
Hyperlipidemia	131 (51.9)	
Hypertension	104 (41.2)	
Coronary artery disease	26 (10.3)	
Hypothyroidism	14 (5.5)	

(Std: standard deviation, min: minimum, max: maximum, F: female, M: male, BNP: brain natriuretic peptide, HDL: high-density lipoprotein, LDL: low-density lipoprotein, AST: aspartate aminotransferase, and ALT: alanine aminotransferase).

**Table 2:** Antidiabetic/antihyperlipidemic medications and BNP levels.

Medications (*n*)		BNP	*p*
**Metformin**	Non-user (61)	49.18 ± 45.69	**0.034**
	User (191)	33.68 ± 36.78	
**SGLT2 inhibitors**	Non-user (110)	42.02 ± 45.49	0.162
	User (142)	33.88 ± 34.08	
**DPP-4 inhibitors**	Non-user (122)	40.47 ± 40.03	0.523
	User (130)	34.58 ± 39.12	
**Pioglitazone**	Non-user (180)	41.46 ± 43.98	**0.021**
	User (72)	27.37 ± 22.85	
**Sulfonylureas**	Non-user (215)	38.12 ± 41.49	0.764
	User (37)	33.42 ± 25.99	
**Insulin**	Non-user (104)	31.44 ± 27.07	0.195
	User (148)	41.64 ± 46.04	
**Statins**	Non-user (165)	40.12 ± 40.33	0.060
	User (87)	32.33 ± 37.85	
**Fibrates**	Non-user (232)	37.84 ± 40.51	0.714
	User (20)	32.70 ± 26.99	

(SGLT2: Sodium–glucose cotransporter-2; DPP-4: dipeptidyl peptidase-4).

**Table 3:** Correlation of BNP levels with clinical data and laboratory parameters.

	R	*p*
**Age**	0.410	**<0.001**
**Diabetes duration**	0.149	**0.018**
**HbA1c**	0.091	0.150
**Fasting glucose**	0.095	0.131
**HDL cholesterol**	0.092	0.145
**LDL cholesterol**	−0.100	0.115
**Triglyceride**	−0.034	0.589
**Urea**	0.072	0.253
**Creatinine**	0.000	0.995
**Hemoglobin**	−0.284	**<0.001**
**ALT**	−0.151	**0.016**
**AST**	−0.044	0.491

**Table 4:** Linear regression models determining factors affecting BNP levels.

Model 1				
	B	S.E.	95% CI for B (Lower–Upper)	*p*
(Constant)	−21.180	15.936	−52.569–10.209	0.185
**Age**	1.305	0.281	0.750–1.859	**<0.001**
**Diabetes duration**	0.078	0.504	−0.915–1.071	0.877
**Male gender**	−12.915	4.653	−22.080–−3.750	**0.006**
**Metformin**	−11.312	5.474	−22.094–−0.530	**0.040**
**Pioglitazone**	−5.486	5.297	−15.920–4.947	0.301
**Model 2**				
	**B**	**S.E.**	**95% CI for B** **(Lower–Upper)**	***p***
(Constant)	31.531	33.886	−35.216–98.279	0.353
**Age**	1.293	0.266	0.769–1.816	**<0.001**
**Metformin**	−11.857	5.948	−23.573–−0.142	**0.047**
**Male gender**	−6.218	5.724	−17.494–5.057	0.278
**HbA1c**	0.738	1.373	−1.966–3.442	0.591
**LDL cholesterol**	−0.040	0.068	−0.174–0.093	0.551
**HDL cholesterol**	0.069	0.209	−0.344–0.481	0.743
**Hemoglobin**	−3.980	1.701	−7.331–−0.628	**0.020**
**Creatinine**	−7.106	12.807	−32.333–18.121	0.580

## Data Availability

The data presented in this study are available on request from the corresponding author.

## Abbreviations

The following abbreviations are used in this manuscript:

BNP	brain natriuretic peptide
CVD	cardiovascular diseases
T2D	type 2 diabetes
AMPK	AMP-dependent kinase
SGLT-2	sodium–glucose cotransporter 2
GLP-1	glucagon-like peptide-1
AST	aspartate aminotransferase
ALT	alanine aminotransferase
HDL	high-density lipoprotein
LDL	low-density lipoprotein
CLIA	chemiluminescence immunoassay
Hb	hemoglobin

## References

[1] Ma C.-X., Ma X.-N., Guan C.-H., Li Y.-D., Mauricio D., Fu S.-B. (2022). Cardiovascular disease in type 2 diabetes mellitus: Progress toward personalized management. Cardiovasc. Diabetol..

[2] Haffner S.M., D’Agostino R., Mykkänen L., Tracy R., Howard B., Rewers M., Selby J., Savage P.J., Saad M.F. (1999). Insulin sensitivity in subjects with type 2 diabetes. Relationship to cardiovascular risk factors: The Insulin Resistance Atherosclerosis Study. Diabetes Care.

[3] Morrish N.J., Wang S.-L., Stevens L.K., Fuller J.H., Keen H. (2001). Mortality and causes of death in the WHO Multinational Study of Vascular Disease in Diabetes. Diabetologia.

[4] Huang P.L. (2009). A comprehensive definition for metabolic syndrome. Dis. Model. Mech..

[5] Palazzuoli A., Iacoviello M. (2023). Diabetes leading to heart failure and heart failure leading to diabetes: Epidemiological and clinical evidence. Heart Fail. Rev..

[6] Sacre J.W., Magliano D.J., Shaw J.E. (2021). Heart failure hospitalisation relative to major atherosclerotic events in type 2 diabetes with versus without chronic kidney disease: A meta-analysis of cardiovascular outcomes trials. Diabetes Metab..

[7] Nadar S.K., Shaikh M.M. (2019). Biomarkers in Routine Heart Failure Clinical Care. Card. Fail. Rev..

[8] Novack M.L., Zubair M. (2023). Natriuretic Peptide B Type Test. StatPearls [Internet].

[9] Pagana K.D., Pagana T.J., Pagana T.N. (2019). Mosby’s Diagnostic & Laboratory Test Reference.

[10] Taylor C.J., Lay-Flurrie S.L., Ordóñez-Mena J.M., Goyder C.R., Jones N.R., Roalfe A.K., Hobbs F.R. (2021). Natriuretic peptide level at heart failure diagnosis and risk of hospitalisation and death in England 2004–2018. Heart.

[11] Tiwari D., Aw T.C. (2024). Emerging Role of Natriuretic Peptides in Diabetes Care: A Brief Review of Pertinent Recent Literature. Diagnostics.

[12] Pop-Busui R., Januzzi J.L., Bruemmer D., Butalia S., Green J.B., Horton W.B., Knight C., Levi M., Rasouli N., Richardson C.R. (2022). Heart Failure: An Underappreciated Complication of Diabetes. A Consensus Report of the American Diabetes Association. Diabetes Care.

[13] Tsai S.-H., Lin Y.-Y., Chu S.-J., Hsu C.-W., Cheng S.-M. (2010). Interpretation and use of natriuretic peptides in non-congestive heart failure settings. Yonsei Med. J..

[14] Herman R., Kravos N.A., Jensterle M., Janež A., Dolžan V. (2022). Metformin and Insulin Resistance: A Review of the Underlying Mechanisms behind Changes in GLUT4-Mediated Glucose Transport. Int. J. Mol. Sci..

[15] Schernthaner G., Brand K., Bailey C.J. (2022). Metformin and the heart: Update on mechanisms of cardiovascular protection with special reference to comorbid type 2 diabetes and heart failure. Metabolism.

[16] Salvatore T., Galiero R., Caturano A., Vetrano E., Rinaldi L., Coviello F., Di Martino A., Albanese G., Marfella R., Sardu C. (2021). Effects of Metformin in Heart Failure: From Pathophysiological Rationale to Clinical Evidence. Biomolecules.

[17] Templer S., Abdo S., Wong T. (2024). Preventing diabetes complications. Intern. Med. J..

[18] Nathan D.M., Bennett P.H., Crandall J.P., Edelstein S.L., Goldberg R.B., Kahn S.E., Knowler W.C., Mather K.J., Mudaliar S., the DPP Research Group (2019). Does diabetes prevention translate into reduced long-term vascular complications of diabetes?. Diabetologia.

[19] Marinescu M., Oprea V.D., Nechita A., Tutunaru D., Nechita L.-C., Romila A. (2023). The Use of Brain Natriuretic Peptide in the Evaluation of Heart Failure in Geriatric Patients. Diagnostics.

[20] Keyzer J.M., Hoffmann J.J., Ringoir L., Nabbe K.C., Widdershoven J.W., Pop V.J. (2014). Age- and gender-specific brain natriuretic peptide (BNP) reference ranges in primary care. Clin. Chem. Lab. Med..

[21] Yan P., Wan Q., Zhang Z., Xu Y., Miao Y., Chen P., Gao C. (2020). Association between Circulating B-Type Natriuretic Peptide and Diabetic Peripheral Neuropathy: A Cross-Sectional Study of a Chinese Type 2 Diabetic Population. J. Diabetes Res..

[22] Bachmann K.N., Huang S., Lee H., Dichtel L.E., Gupta D.K., Burnett J.C., Miller K.K., Wang T.J., Finkelstein J.S. (2019). Effect of Testosterone on Natriuretic Peptide Levels. J. Am. Coll. Cardiol..

[23] Cediel G., Codina P., Spitaleri G., Domingo M., Santiago-Vacas E., Lupón J., Bayes-Genis A. (2021). Gender-Related Differences in Heart Failure Biomarkers. Front. Cardiovasc. Med..

[24] Maffei S., Del Ry S., Prontera C., Clerico A. (2001). Increase in circulating levels of cardiac natriuretic peptides after hormone replacement therapy in postmenopausal women. Clin. Sci..

[25] Knudsen C.W., Vik-Mo H., Omland T. (2005). Blood haemoglobin is an independent predictor of B-type natriuretic peptide (BNP). Clin. Sci..

[26] Karakoyun I., Colak A., Arslan F.D., Hasturk A.G., Duman C. (2017). Anemia considerations when assessing natriuretic peptide levels in ED patients. Am. J. Emerg. Med..

[27] He W.-T., Mori M., Yu X.-F., Kanda T. (2016). Higher BNP levels within physiological range correlate with beneficial nonfasting lipid profiles in the elderly: A cross-sectional study. Lipids Health Dis..

[28] Srisawasdi P., Vanavanan S., Charoenpanichkit C., Kroll M.H. (2010). The effect of renal dysfunction on BNP, NT-proBNP, and their ratio. Am. J. Clin. Pathol..

[29] Vickery S., Price C.P., John R.I., Abbas N.A., Webb M.C., Kempson M.E., Lamb E.J. (2005). B-type natriuretic peptide (BNP) and amino-terminal proBNP in patients with CKD: Relationship to renal function and left ventricular hypertrophy. Am. J. Kidney Dis..

[30] Khan A.M., Cheng S., Magnusson M., Larson M.G., Newton-Cheh C., McCabe E.L., Coviello A.D., Florez J.C., Fox C.S., Levy D. (2011). Cardiac natriuretic peptides, obesity, and insulin resistance: Evidence from two community-based studies. J. Clin. Endocrinol. Metab..

[31] Chang H.-R., Hsieh J.-C., Chen M.Y.-C., Wang J.-H., Hsu B.-G., Wang L.-Y. (2014). N-terminal pro-B-type natriuretic peptide is inversely associated with metabolic syndrome in hypertensive patients. Am. J. Med. Sci..

[32] Tsukamoto O., Fujita M., Kato M., Yamazaki S., Asano Y., Ogai A., Okazaki H., Asai M., Nagamachi Y., Maeda N. (2009). Natriuretic peptides enhance the production of adiponectin in human adipocytes and in patients with chronic heart failure. J. Am. Coll. Cardiol..

[33] Mezzasoma L., Talesa V.N., Romani R., Bellezza I. (2020). ANP and BNP Exert Anti-Inflammatory Action via NPR-1/cGMP Axis by Interfering with Canonical, Non-Canonical, and Alternative Routes of Inflammasome Activation in Human THP1 Cells. Int. J. Mol. Sci..

[34] Chen J., Jiang C., Guo M., Zeng Y., Jiang Z., Zhang D., Tu M., Tan X., Yan P., Xu X. (2024). Effects of SGLT2 inhibitors on cardiac function and health status in chronic heart failure: A systematic review and meta-analysis. Cardiovasc. Diabetol..

[35] Lundin M., Ferrannini G., Mellbin L., Johansson I., Norhammar A., Näsman P., Shahim B., Smetana S., Venkateshvaran A., Wang A. (2022). SOdium-glucose CO-transporter inhibition in patients with newly detected Glucose Abnormalities and a recent Myocardial Infarction (SOCOGAMI). Diabetes Res. Clin. Pract..

[36] Shimizu W., Kubota Y., Hoshika Y., Mozawa K., Tara S., Tokita Y., Yodogawa K., Iwasaki Y.-K., Yamamoto T., Takano H. (2020). Effects of empagliflozin versus placebo on cardiac sympathetic activity in acute myocardial infarction patients with type 2 diabetes mellitus: The EMBODY trial. Cardiovasc. Diabetol..

[37] Zinman B., Wanner C., Lachin J.M., Fitchett D., Bluhmki E., Hantel S., Mattheus M., Devins T., Johansen O.E., Woerle H.J. (2015). Empagliflozin, Cardiovascular Outcomes, and Mortality in Type 2 Diabetes. N. Engl. J. Med..

[38] McMurray J.J.V., Solomon S.D., Inzucchi S.E., Køber L., Kosiborod M.N., Martinez F.A., Ponikowski P., Sabatine M.S., Anand I.S., Bělohlávek J. (2019). Dapagliflozin in Patients with Heart Failure and Reduced Ejection Fraction. N. Engl. J. Med..

[39] Dorkhan M., Frid A., Groop L. (2008). Differences in effects of insulin glargine or pioglitazone added to oral anti-diabetic therapy in patients with type 2 diabetes: What to add—Insulin glargine or pioglitazone?. Diabetes Res. Clin. Pract..

[40] Tahrani A.A., Varughese G.I., Scarpello J.H., Hanna F.W. (2007). Metformin, heart failure, and lactic acidosis: Is metformin absolutely contraindicated?. BMJ.

[41] Top W.M.C., Lehert P., Schalkwijk C.G., Stehouwer C.D.A., Kooy A. (2021). Metformin and N-terminal pro B-type natriuretic peptide in type 2 diabetes patients, a post-hoc analysis of a randomized controlled trial. PLoS ONE.

[42] Fawzi H.A., Sabbar R., Kadhim S.A.A., Flayih A., Mohammad B., Swadi A. (2023). Metformin effects on cardiac parameters in non-diabetic Iraqi patients with heart failure and mid-range ejection fraction—A comparative two-arm parallel clinical study. J. Med. Life.

[43] Sokolova L., Belchina Y., Cherviakova S., Vatseba T., Kovzun O., Pushkarev V. (2020). The effect of metformin treatment on the level of GLP-1, NT-proBNP and endothelin-1 in patients with type 2 diabetes mellitus. Int. J. Endocrinol..

